# Supplementary material for: Gender Composition in Occupational Therapy Journals’ Editorial Boards
Source: Int J Environ Res Public Health. 2023 Feb 16;20(4):3458. doi: 10.3390/ijerph20043458 (PMC9966705; doi:10.3390/ijerph20043458)
Supplement: Supplementary file 1 [file ijerph-20-03458-s001.zip › ijerph-2195208-supplementary.pdf]

Table S1. Journals information

Rank	Title	EBM Total	Men	%	Women	%	Editorial Leader (El)	Men (El)	Women (El)	Associate Editor (AE)	Men (AE)	Women (AE)	Editorial Board Member (EBM)	Man (EBM)	Women (EBM)	Type		SJR	SJR Quartile	H index	Country	Publisher	Coverage	Categories
2	American Journal of Occupational Therapy	19	6	31.5o	13	68.42	1	0	1	15	5	10	3	1	2	journal	2246	0.657	Q1	87	United States	American Occupational Therapy Association, Inc	1947-2021	Medicine (miscellaneous); Occupational Therapy
4	Australian Occupational Therapy Journal	10	2	20	8	80	1	0	1	4	1	3	5	1	4	journal	1856	0.519	Q1	47	United Kingdom	Wiley-Blackwell Publishing Ltd	1952-1953, 1956-2021	Occupational Therapy
11	Brazilian Journal of Occupational Therapy	55	11	20	44	80	1	0	1	1	0	1	53	11	42	journal	0.13 (JCI)	0.318	Q3	7	Brazil	Universidade Federal de Sao Carlos	2018-2021	Education; Health (social science); Occupational Therapy
8	British Journal of Occupational Therapy	14	5	35.71	9	64.2	1	1	0	5	1	4	8	3	5	journal	1243	0.372	Q2	49	United Kingdom	SAGE Publications Inc.	1974-1976, 1996-2021	Occupational Therapy
6	Canadian Journal of Occupational Therapy	9	2	22.22	7	77.78	2	0	2	7	2	5	0	0	0	journal	1614	0.405	Q2	58	United States	SAGE Publications Inc.	1933-2021	Occupational Therapy
14	Hong Kong Journal of Occupational Therapy	50	29	58	21	42	1	1	0	4	3	1	45	25	20	journal	0.917	0.21	Q3	15	Singapore	Elsevier (Singapore) Pte Ltd	2002-2021	Occupational Therapy
15	Irish Journal of Occupational Therapy	16	4	25	12	75	1	0	1	5	0	5	10	4	6	journal	Not found	0.17	Q4	4	United Kingdom	Emerald Group Publishing Ltd.	2018-2021	Occupational Therapy; Public Health, Environmental and Occupational Health
2	Journal of Occupational Rehabilitation	43	24	55.81	19	44.19	1	1	0	2	0	2	40	23	17	journal	3489	1.049	Q1	76	United States	Springer New York	1991-2021	Occupational Therapy; Rehabilitation
12	Journal of Occupational Therapy, Schools, and Early Intervention	37	3	8.11	34	91.89	1	0	1	0	0	0	36	3	33	journal	0.22 (JCI)	0.262	Q3	12	United Kingdom	Routledge	2008-2021	Education; Occupational Therapy; Social Psychology
5	Journal of Vocational Rehabilitation	118	62	52.54	56	47.46	1	1	0	0	0	0	117	61	56	journal	0.41(JCI)	0.409	Q1	40	Netherlands	IOS Press BV	1991-2021	Occupational Therapy; Rehabilitation
10	Occupational Therapy in Health Care	33	8	24.24	25	75.76	1	0	1	3	1	2	29	7	22	journal	0.53 (JCI)	0.33	Q2	26	United States	Informa Healthcare	1984-1993, 1995-2021	Medicine (miscellaneous); Occupational Therapy
16	Occupational Therapy in Mental Health	66	8	12.12	58	87.88	2	0	2	1	0	1	63	8	55	journal	0.32 (JCI)	0.289	Q3	20	United States	Routledge	1980, 1982-1993, 1995-2021	Applied Psychology (Q3); Psychiatry and Mental Health (Q3); Public Health, Environmental and Occupational Health (Q3)
9	Occupational Therapy International	17	5	29.41	12	70.59	1	0	1	2	0	2	14	5	9	journal	1448	0.354	Q2	39	Egypt	Hindawi Limited	1994-2021	Medicine (miscellaneous); Occupational Therapy

7	OTJR Occupation, Participation and Health	18	5	27.78	13	72.22	1	1	0	16	4	12	1	0	1	journal	0.378	0.402	Q2	42	United States	SAGE Publications Inc.	2002-2021	Occupational Therapy
13	Physical and Occupational Therapy in Pediatrics	110	14	12.73	96	87.27	2	1	1	3	0	3	105	13	92	journal	0.26 (JCI)	0.254	Q3	21	United States	Informa Healthcare	1980-2021	Geriatrics and Gerontology; Gerontology; Occupational Therapy; Rehabilitation
13	Physical and Occupational Therapy in Geriatrics	26	15	57.69	11	42.31	1	1	0	2	1	1	23	13	10	journal	0.26 (JCI)	0.254	Q3	21	United States	Informa Healthcare	1980-2021	Geriatrics and Gerontology; Gerontology; Occupational Therapy; Rehabilitation
17	Scandinavian Journal of Occupational Therapy	26	4	15.3	22	84.62	1	0	1	0	0	0	25	4	21	journal	2611	0.579	Q2	44	United Kingdom	Taylor and Francis Ltd.	1994-1996, 1999-2021	Public Health, Environmental and Occupational Health (Q2)

Table S2. Journals indexes

Rank	Title	JCR	SJR	SJR Quartile	h-index	EB Total	
2	American Journal of Occupational Therapy	2.246	0.657	Q1	87	19	2.85%
4	Australian Occupational Therapy Journal	1,856	0.519	Q1	47	10	1.50%
11	Brazilian Journal of Occupational Therapy	0.13 (JCI)	0.318	Q3	7	55	8.25%
8	British Journal of Occupational Therapy	1.243	0.372	Q2	49	14	2.10%
6	Canadian Journal of Occupational Therapy	1.614	0.405	Q2	58	9	1.35%
14	Hong Kong Journal of Occupational Therapy	0.917	0.21	Q3	15	50	7.50%
15	Irish Journal of Occupational Therapy	Not found	0.17	Q4	4	16	2.40%
1	Journal of Occupational Rehabilitation	3.489	1.049	Q1	76	43	6.45%
12	Journal of Occupational Therapy, Schools, and Early Intervention	0.22 (JCI)	0.262	Q3	12	37	5.55%
5	Journal of Vocational Rehabilitation	0.41(JCI)	0.409	Q1	40	118	17.69%
10	Occupational Therapy in Health Care	0.53 (JCI)	0.33	Q2	26	33	4.95%
16	Occupational Therapy in Mental Health	0.32 (JCI)				66	9.90%
9	Occupational Therapy International	1,448	0.354	Q2	39	17	2.55%
7	OTJR Occupation, Participation and Health	0.378	0.402	Q2	42	18	2.70%
13	Physical and Occupational Therapy in Geriatrics	0.26 (JCI)	0.254	Q3	21	110	16.49%
3	Physical and Occupational Therapy in Pediatrics	2,360	0.527	Q1	48	26	3.90%
17	Scandinavian Journal of Occupational Therapy	2,611				26	3.90%
						667	100%

Table S3. Men-Women Ratio.

Title	Title	EBMs total	n	%	n	%	M-W Ratio
Journal of Occupational Therapy, Schools, and Early Intervention	JOTSEI	37	34	91.89%	3	8.11%	11.33
Occupational Therapy in Mental Health	OTMH	66	58	87.88%	8	12.12%	7.25
Physical and Occupational Therapy in Geriatrics	POTG	110	96	87.27%	14	12.73%	6.86
Scandinavian Journal of Occupational Therapy	SJOT	26	22	84.62%	4	15.38%	5.50
Australian Occupational Therapy Journal	AUSOTJ	10	8	80.00%	2	20.00%	4.00
Brazilian Journal of Occupational Therapy	CADBTO	55	44	80.00%	11	20.00%	4.00
Canadian Journal of Occupational Therapy	CJOT	9	7	77.78%	2	22.22%	3.50
Occupational Therapy in Health Care	OTHC	33	25	75.76%	8	24.24%	3.13
Irish Journal of Occupational Therapy	IJOT	16	12	75.00%	4	25.00%	3.00
OTJR Occupation, Participation and Health	OTJR	18	13	72.22%	5	27.78%	2.60
Occupational Therapy International	OTI	17	12	70.59%	5	29.41%	2.40
American Journal of Occupational Therapy	AJOT	19	13	68.42%	6	31.58%	2.17
British Journal of Occupational Therapy	BJOT	14	9	64.29%	5	35.71%	1.80
Journal of Vocational Rehabilitation	JVR	118	56	47.46%	62	52.54%	0.90
Journal of Occupational Rehabilitation	JOR	43	19	44.19%	24	55.81%	0.79
Physical and Occupational Therapy in Pediatrics	POTP	26	11	42.31%	15	57.69%	0.73
Hong Kong Journal of Occupational Therapy	HKJOT	50	21	42.00%	29	58.00%	0.72
